# Silent dissemination of HTLV-1: evidence of intrafamilial transmission in a Brazilian reference centre

**DOI:** 10.1590/0074-02760240191

**Published:** 2025-03-31

**Authors:** Daniele Leite Alves, Roberta Muniz Luz Silva, João Pedro Melo de Freitas Santos, Rebeca Leão Amorim, Carolina Souza Santana, Felipe de Oliveira Andrade, Saadia Oliveira Ribeiro, Giselle Calasans de Souza Costa, Luciane Amorim Santos, Davi Tanajura Costa, Fernanda Khouri Barreto

**Affiliations:** 1 Universidade Federal da Bahia, Instituto Multidisciplinar em Saúde, Vitória da Conquista, BA, Brasil; 2 Laboratório Central de Vitória da Conquista, Vitória da Conquista, BA, Brasil; 3 Centro de Suporte e Apoio à Vida, Vitória da Conquista, BA, Brasil; 4 Universidade Federal da Bahia, Instituto de Ciências de Saúde, Salvador, BA, Brasil; 5 Fundação Oswaldo Cruz-Fiocruz, Instituto Gonçalo Moniz, Salvador, BA, Brasil; 6 Escola Bahiana de Medicina e Saúde Pública, Salvador, BA, Brasil; 7 Universidade Estadual do Sudoeste da Bahia, Vitória da Conquista, BA, Brasil

**Keywords:** human T-lymphotropic virus type 1, family cluster, mother-to-child transmission, genetics in primary care, public health, access to diagnosis

## Abstract

**BACKGROUND:**

The HTLV-1 affects 5 to 10 million people worldwide. It is estimated that 5 to 10% of the infected individuals develop severe diseases, such as HTLV-Associated Myelopathy/Tropical Spastic Paraparesis (HAM/TSP) or Adult T-Cell Leukaemia/Lymphoma (ATLL). HTLV-1 transmission can occur mainly through unprotected sexual contact and from mother to child during breastfeeding. No vaccines can contain this infection, and strategies to prevent transmission become a priority. Therefore, it is important to know the main dissemination routes of each region to design the best public health strategies for controlling the spread of this virus.

**OBJECTIVES:**

This study aimed to evaluate the prevalence of family aggregation in HTLV-1 infection among patients treated at a reference centre in Brazil.

**METHODS:**

A cross-sectional study was conducted with patients between July 2021 and August 2022. A total of 67 individuals were attended, of which 17 were classified as index cases due to a history of family aggregation, with 120 family contacts.

**FINDINGS:**

We found a prevalence of 36% of individuals positive for HTLV-1 and the same for HTLV-1 negative, while 28% of the family members had unknown serology. The possible transmission routes were identified, and the familial transmission histories within each family were hypothesised.

**MAIN CONCLUSIONS:**

These data can support specific decisions regarding the local reality, such as a better health strategy, especially in preventing new HTLV-1 cases.

The human T-lymphotropic virus type 1 (HTLV-1) affects 5 to 10 million people worldwide. Southwestern Japan, Africa, the Caribbean, and the Middle East, Australia, and South America are considered endemic regions.^1^ In Brazil, the country’s Northeast region, especially Bahia State, presents a high prevalence of this infection.^2^ In 2003 it was shown that 1.8% of the general population of Salvador, the capital of Bahia, was infected by HTLV-1.^3^ More recently, an epidemiological study revealed that Bahia has the highest infection rate in Brazil, with the capital city of Salvador standing out with 39.3% of the state HTLV-1 cases, followed by the city of Vitória da Conquista, which shows a prevalence of 10% of the cases.^4^

HTLV-1 may cause multiple complications, among which the most severe diseases are HTLV-1-associated myelopathy/tropical spastic paraparesis (HAM/TSP) and adult T-cell leukaemia/lymphoma (ATLL), which are diseases with high morbidity and mortality.^5^ Although the majority of people living with HTLV-1 (PLWH) are frequently classified as asymptomatic carriers (AC), a considerable portion of these patients report nonspecific symptoms, such as intense muscle pain, urinary incontinence, and other clinical conditions such as depression and other emotional and psychosocial factors that negatively impact the quality of infected individuals.^6^ Considering that the HTLV-1-associated diseases manifest itself slowly and progressively, people infected by HTLV-1 remain exposed to the virus for long periods until present some signs or symptoms.^7^ In this sense, asymptomatic and subclinical HTLV-1 infection facilitates the maintenance and spread of this virus within communities.^8^

Mother-to-child transmission (MTCT) of HTLV-1 through prolonged breastfeeding is considered the primary route of transmission, with unprotected sexual contact.^9^ Infection through MTCT is particularly concerning due to its exceptionally high risk for infants to develop ATLL during their lives, as well as is associated with the high proviral load (PVL).^10^ ^,^ ^11^

The phenomenon outlined by the distribution of signs, symptoms, and complications in some family nuclei is known as “Familial Aggregation”.^12^ Recently, a study performed by the Bahia School of Medicine and Public Health’s Integrative Multidisciplinary HTLV Centre (CHTLV) demonstrated a prevalence of 53.5% for familial aggregation among HTLV-1 infection.^13^ In addition, a recent systematic review highlighted the importance of this topic within the HTLV-1 field. It emphasises that investigating the familial aggregation phenomenon presents an optimistic potential for a better understanding and prevention of certain diseases, especially HTLV-1 infection. This approach may contribute to unravelling the puzzle of the wide and idiosyncratic magnitude of symptoms, complications, and the severity of health conditions associated with HTLV-1.^12^

Therefore, knowing the prevalence of the familial aggregation phenomenon and its main possible transmission routes is fundamental to implementing effective actions to combat infection, strengthening public health strategies to reduce HTLV-1 transmission. Thus, this study aimed to evaluate the prevalence of familial aggregation and identify the main possible HTLV-1 transmission routes among patients treated at a reference centre in Vitória da Conquista, Bahia, Brazil.

## SUBJECTS AND METHODS

*Study design and index case identification* - This study uses a cross-sectional and descriptive evaluation of data obtained from medical records and clinical consultations of HTLV-1 carriers registered in the specialised HTLV care service (SAE/HTLV) at Centro de Apoio e Atenção à Vida (CAAV) from July 2021 to August 2022.

Patient care was divided into three stages: (1) interview for collecting epidemiological and social data through structured questionnaires created using the KoboToolbox platform by the researchers (see Supplementary data);^14^ (2) clinical evaluation by a neurologist to evaluate neurological symptoms associated with HTLV-1 infection; (3) analysis of the medical records to search for more members of the family who tested positive for HTLV-1, configuring a possible case of family aggregation. The inclusion criteria comprised patients aged 12 years or older, with at least one family member testing positive for HTLV-1, and those who had at least one consultation with the service’s neurologist throughout the study period. The exclusion criterion was individuals with cognitive deficits and/or psychiatric disorders that prevented them from responding to questionnaires.

*Data analysis* - All people living with HTLV-1 (PLWH) followed at CAAV from July 2021 to August 2022 were considered a possible index case for the familial aggregation phenomenon. The CAAV medical records have specific fields for collecting data on family relationships. We analyse this data to construct possible patient-family relationships, crossing the data from these medical records. To avoid two or more index cases in the same family, we use the criterion of who was seen first at the service during the period of the study (information obtained from the patient’s medical records). To identify and characterise the possible routes of intrafamily transmission for each index case, the family data available in medical records were evaluated in search of first-degree relatives with serological testing for HTLV-1. The following kinship degrees were considered:

(a) Mothers of the index cases;

(b) Sons and daughters of the female index cases;

(c) Spouses with stable relationships of index cases;

(d) Siblings, parents (male), and grandparents only in cases of serologic positivity of the mothers of index cases;

(e) Sons and daughters of female spouses of index cases with positive serology.

The epidemiological, social, and clinical data were tabulated and analysed using Microsoft Office Excel^®^.^15^ Through analysis of data on patient-family relationships collected in medical records, the family heredograms were generated to infer likely routes of HTLV-1 transmission between family members using the Invitae family history tool, version 2.0.^16^ To determine the prevalence of intrafamily transmission, it was considered the total of indicative cases and their respective communicating families. Prisma GraphPad program was used for the statistical analyses, and significance was assumed when p < 0.05.^17^

*Ethical issues* - All research participants were thoroughly informed about the procedures and conduct regarding collecting information and the research objectives. Individuals over 18 years of age who agreed to participate in the research signed an informed consent form (ICF) authorising the use of their data, and those between 12 and 18 years of age were also given the ICF from the parent/guardian. This study received approval from the Human Research Ethics Committee of the Instituto Multidisciplinar em Saúde at the Universidade Federal da Bahia (CEP/IMS/UFBA), with protocol number 5.064.102.

## RESULTS

**Fig. 1:**
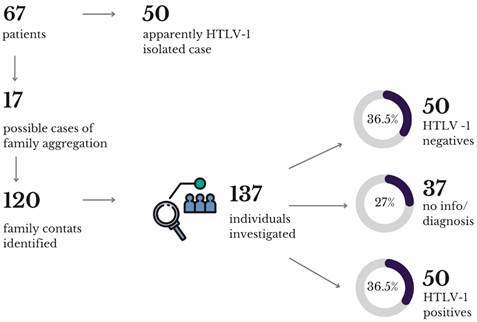
flowchart of the study. Created with Canva.

*General and clinical characteristics* - A total of 67 patients were attended at CAAV from July 2021 to August 2022. The median age was 49 years, with patients’ ages ranging from 20 to 84 years. There were more females (79.1%) than males (20.9%), and the majority of participants self-identified as black, indigenous, and people of colour (BIPOC) (79.1%), while the white population constituted 20.9%.^18^ During this period, we identified 17 index cases due to a history of possible familial aggregation, resulting in 120 family contacts identified after medical records evaluation (Fig. 1).

**Fig. 2:**
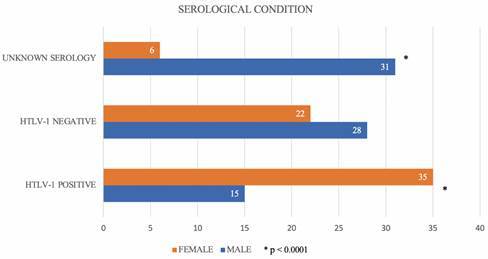
serological profile by gender of HTLV-1 families. Chi-square test.

From the total of 137 individuals investigated (17 index cases + 120 family contacts), the prevalence of HTLV-1 in the family nucleus of the study population evaluated was 36,5%, as well as the HTLV-1 negative patients. Furthermore, 27% of the individuals investigated were not tested or had no information about the HTLV-1 infection in the medical records. It was also observed that more men (n = 31) had unknown serological status than women (n = 6). Inversely, a greater number of reactive HTLV-1 cases were found in women (n = 35) than in men (n = 15). These gender analyses showed a significant difference through the Chi-Square Test (p < 0.0001) (Fig. 2).

Among all index cases, 47.1% were classified as positive for HAM/TSP through neurological examination. Of the 52.9% of patients who did not meet the criteria to be classified as HAM/TSP, a significant proportion (55.5%) presented multisystemic complaints interpreted as nonspecific symptoms, including neurological symptoms (22.2%), rheumatic symptoms (11.1%), genitourinary symptoms (11.1%), and gastrointestinal symptoms (11.1%). Within this set of symptoms, 44.4% were necessarily associated with painful events, and 44.5% of non-HAM index patients had no complaints and were considered asymptomatic.

Analysing only the index patients classified as HAM/TSP, some other clinically relevant findings were observed, such as depression (12.5%), insomnia (12.5%), and recurrent urinary tract infection (12.5%), in addition to the symptoms of weakness in the lower limbs, lower back pain radiating to the lower limbs, and urinary incontinence characteristic of the pyramidal syndrome and overactive bladder syndrome that constitute HAM/TSP.

Regarding the symptomatology of family contacts, 9.1% were diagnosed with HAM/TSP, while 27.3% (n = 9) were non-HAM/TSP. Unfortunately, we did not obtain information about the neurological condition of 60.6% (n = 20).

**Fig. 3:**
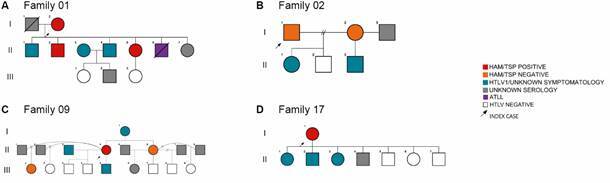
heredogram showing a possible family aggregation case of HTLV-1 infection - Families 01, 02, 09 and 17.

*Possible intrafamilial transmission routes* - The analysis of the 17 constructed heredograms showed some interesting results regarding the HTLV-1 aggregation phenomenon. From the 50 infected individuals, probable vertical transmission occurred in 54% of cases (27/50), and probable sexual transmission occurred in 14% (7/50). Ten families were affected by vertical transmission (Families 03, 04, 05, 08, 10, 12, 14, 15, 16, and 17), three by sexual transmission (Families 07, 11, and 13), and four by both vertical and sexual transmission (Families 01, 02, 06, and 09). We highlighted here four families due to the complexity of possible transmissions and the number of family members affected by HTLV-1 (Fig. 3).

The analysis of Family 01 suggested that a female index case (I-2) was the starting point for the confirmed infection of 6 relatives (II-1, 2, 3, 4, 5, and 6) and 3 other possible infections (I-1, II-7, III-2). The probable flow of vertical transmission was evidenced in communicants II-1, 2, 4, 5, and 6, and probable sexual transmission in communicant II-3. The only communicants with a confirmed non-reactive serology were III-1 and III-3. Among the patients reactive for HTLV-1 (n = 7), three have a clinical diagnosis of HAM/TSP (I-2, II- 2 and 5), three have HTLV-1 with unknown symptomatology (II-1, 3 and 4), and one had a diagnosis of ATLL before death (II-6) (Fig. 3A).

In Family 02 there were at least three other individuals infected with HTLV-1 from the index case (I-1). The index case was male, and the probable initial transmission route was sexual contact (I-1 and I-2); these individuals do not have a diagnosis of HAM/TSP. The probable flow of vertical transmission was evidenced in communicants II-1 and II-3. The only communicant with a confirmed non-reactive serology is communicant II-2. Communicant I-3 does not have known serology (Fig. 3B).

The index case II-4, who has HAM/TSP, led to the discovery of five more cases of HTLV-1 infection among members of Family 09. These include the partner (II-3), children (III-1 and III-5), mother (I-1), and sister (II-6). Although the index case is patient II-4, the transmission route possibly started with individual I-1 or earlier. The serology of the sexual contacts from past relationships (II-1, II-2, II-7, and II-8) is unknown. Possible sexual transmission was evidenced between II-3 and II-4 (Fig. 3C).

In Family 17, we found strong evidence of vertical transmission since three individuals (II-1, II-2, and II-3) were infected with HTLV-1 besides the female index case I-1 (mother). Individual II-4 has unknown serology, and the other contacts (II-5, II-6, and II-7) do not have HTLV-1 infection (Fig. 3D).

**Fig. 4:**
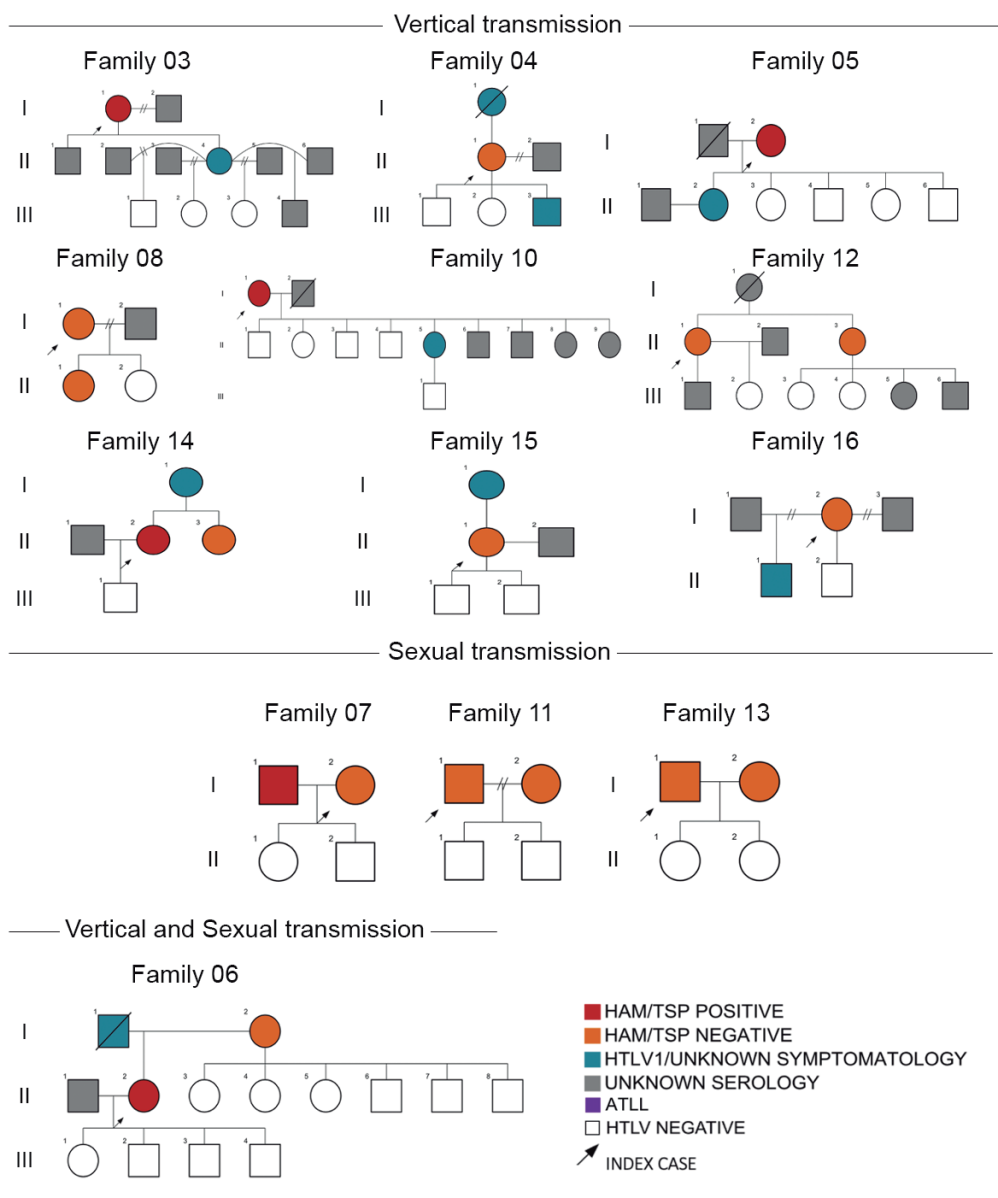
representation of 13 families with each probable HTLV-1 transmission routes based on epidemiological.

Additionally, the other 13 families studied are represented in Fig. 4, in which nine of them have possible cases of vertical transmission, and three had cases of sexual transmission. Both routes of viral transmission were observed in Family 6. It is worth mentioning that Family 12 was classified as having a vertical transmission profile due to information contained in the medical record, where the index case reports that its mother possibly had HAM/TSP and became a wheelchair user in adulthood.

## DISCUSSION

Families with several HTLV-1 cases have been reported worldwide since the discovery of this virus.^19^ ^,^ ^20^ A systematic review recently showed that Japan and Brazil are the countries that publish the most data on familial aggregation of HTLV-1, demonstrating their interest in understanding this phenomenon.^12^ These Brazilian studies have been carried out in different geographic regions to reveal this phenomenon’s real epidemiological situation. Recently, a study performed by Botelho et al., showed that HTLV-1 is transmitted silently between *quilombola* communities of Pará, Brazil.^21^ Another study in the same Brazilian state revealed a prevalence of 43.5% of HTLV-1 in families, which reinforces the need to investigate the familial aggregation of this virus.^22^

Another important study was conducted in Bahia, considered an endemic Brazilian state, and estimated an HTLV-1 prevalence of 32.9% in family members.^19^ Here, we observed a prevalence of this phenomenon in 25.4% of the cases (n = 17). Analysing the communicating patients, in a sample size of 137 individuals, 50 are infected, corroborating with these authors that suggested that HTLV-1 can affect several members of the same family. Our study is the first that demonstrated the prevalence of the silent dissemination of HTLV-1 in a reference centre of Vitória da Conquista, one of the largest cities in northeastern Brazil. The importance of this subject was highlighted by Rosadas et al., who evidenced Brazil’s main HTLV-1 transmission routes and suggested public health strategies to try to block them.^8^

It is important to highlight that the heterogeneous distribution of the HTLV-1 infection and the scarcity of data suggest much higher numbers of affected families. In this sense, a study with Japanese immigrants and their descendants residing in a non-endemic region of central Brazil, investigated 6 families, of which five had at least one case of HTLV-1, demonstrating a prevalence of 22% of infected family members.^23^ Another study conducted in a non-endemic location in Argentina reported four members of the same family positive for HTLV-1, all of which presented the same LTR, tax, and env sequences.^24^ In a non-endemic region of China, 11 families were reported, of which six showed a possible horizontal transmission.^25^ All these studies, like the present one, aim to make intrafamilial transmission of HTLV-1 visible to strengthen preventive medicine and corroborate with the public call for action to eradicate the virus proposed by the World Health Organization (WHO).^26^

In our study, as expected, the HTLV-1 prevalence in women was higher than in men (70%). Although the higher efficiency of sexual transmission from men to women is established, probably due to contact of viral particles on the vaginal mucosa, it is also important to consider that women use more health care services than men.^27^ However, the point is that this high virus prevalence in women can facilitate vertical transmission through breastfeeding, directly impacting the phenomenon of family clustering, as observed in this study. In Bahia, prenatal screening is mandatory and in Brazil breastfeeding is contraindicated for mothers living with the virus.^28^ ^,^ ^29^ Unfortunately, in some cases, health professionals are often not adequately trained to guide the patients and due to lack of instruction, some infected women breastfeed. In this sense, this study highlights the importance of education associated with public health measures.

Our data demonstrated that vertical transmission occurred more frequently than horizontal transmission (58.82% of families). The primary described route of mother-to-child transmission is through breastfeeding, and this high rate of possible vertical transmission may be related to the failure of preventive measures, such as the cessation of breastfeeding in the postnatal period, once most infected women were not aware of their infection.^11^ ^,^ ^30^ Another important question is on the risk factors for in-utero peripartum transmission since little is known about the efficacy of a caesarean to reduce this possible HTLV-1 route.^9^ ^,^ ^11^ Regarding sexual transmission, prevention measures such as the free distribution of condoms and health education policies can suppress this type of transmission, provided that the individual is aware of their serological status. It is important to highlight that implementing antenatal screening and a program to prevent vertical transmission in Bahia may justify the lack of vertical transmission in families with only horizontal transmission routes.

Here, we observed that more men (n = 31) had unknown serological status than women. Although a laboratory test is sufficient to carry out the serological screening of HTLV-1 infection, the early diagnosis and information about this disease are still very limited. Most people infected with this retrovirus only discover the diagnosis long after the onset of symptoms by the long latency period of the infection and by the absence of specific symptoms in the majority of infected people.^6^ ^,^ ^7^ Therefore, many family members of people living with HTLV-1 infection in the world remain undiagnosed.

In the face of this, it is important to emphasise that those individuals who have nonspecific symptoms or are considered asymptomatic and/or unaware that they are infected with HTLV-1 continue to transmit the virus, often in the family environment. Probably, this is the major cause of HTLV-1 familial aggregation.

Although it is not fully established if the type of transmission could influence the HTLV-1 outcome, it is noted that several serious clinical manifestations caused by HTLV-1 infection seem to be associated with some routes. Rosadas et al., show that infections at the beginning of life may be associated with a higher risk of developing diseases with high morbidity and mortality.^11^ Silva et al., identified families with clustering of infective dermatitis associated with HTLV-1 (IDH) and/or HAM/TSP and suggested that a high quantity of HTLV-1 in the milk could increase the chance of development of HAM/TSP in breast-fed children.^31^ In Rio de Janeiro, nine cases with family aggregation and associated diseases such as ATLL and HAM/TSP were identified, suggesting an exploratory analysis to investigate the relationship between immunogenetic profile and proviral load to determine the risk of associated pathologies.^32^

In our study, 22% of the HTLV-1 patients were diagnosed with HAM/TSP. Out of the 17 families analysed here, only one family had more than one case of HAM/TSP (three cases). Significant clinical profile similarities were not found within the families evaluated in this study. A study that yielded similar results suggests that HAM/TSP generally affects separate individuals, but there are specific peculiarities that can lead to clusters of HAM/TSP within families without a distinct pattern.^33^ In another study, the nucleotide sequences of HTLV-1 proviruses in three family members who were positive for HAM/TSP were described, and similar mutations were observed in all three individuals.^34^ More molecular data are needed to understand possible HTLV-1 genetic signatures and/or biomarkers of disease progression.

Although here we describe 17 HTLV-1 family aggregation cases, we highlighted that other family connections that may exist are not described in this study, considering that the family relationship history was constructed from the analysis of the patient’s medical records and interviews, making it possible that other relationships were not captured. It is worth noting that in our data 37% were not tested and although we hypothesised vertical transmission as the main route of infection, maybe some families could have an alternative route. Furthermore, performing molecular analysis to identify possible genetic signatures from the LTR region of each family member may help in confirming the transmission rote by phylogenetic assessment strategy.

*In conclusion* - Controlling infectious diseases becomes increasingly important as the world moves toward preventive medicine. As no prophylactic or therapeutic vaccines can contain HTLV-1 infection, interrupting the virus transmission becomes the main choice. In this work, it was possible to observe families with several cases of HTLV-1, highlighting the silent spread of this virus, mainly caused by the lack of knowledge of the infection.

The HTLV-1 infection can be devastating, and when it affects families, the outcome becomes even more disquieting. Therefore, this work reinforced that interrupting the virus transmission in the family environment is perhaps the main way to eliminate HTLV-1. Our data demonstrates the need to expand general testing, with priority on population groups at high risk of infection, such as familiars. In addition, we highlight the importance of counselling people living with HTLV-1. The implementation of public policies and investment in HTLV-1 studies in this direction might play a key role in limiting intrafamilial transmission.
