# Supplementary material for: Silent dissemination of HTLV-1: evidence of intrafamilial transmission in a Brazilian reference centre
Source: Mem Inst Oswaldo Cruz. 2025 Mar 31;120:e240191. doi: 10.1590/0074-02760240191 (PMC11964092; doi:10.1590/0074-02760240191)
Supplement: Supplementary file 1 [file 1678-8060-mioc-120-e240191-s.pdf]

Social Questionnaire – HTLV-1

Interview date
YYYY-MM-DD
Patient identification number:
First and last name:
Social name:

Age
a) 12 to 39 years
b) 40 to 59 years
c) 60 years or older
d) Not informed

Date of birth
YYYY-MM-DD

Nacionality:

Gender
a) Male Cisgender
b) Male Transgender
c) Female Cisgender
d) Male Cisgender
e) Not Provided
f) Others

If (f): What identify?

Ethnicity
Self-declaration
a) White black
b) Brown
c) Yellow
d) Indigenous
e) Not informed

Marital status
a) Stable relationship
b) Casual relationships
c) Single widowed
d) Not informed

Sexual orientation
a) Heterosexual
b) Homosexual
c) Bisexual
d) Other
e) Not informed

If (e): What sexual orientation?

Profession
a) Housewife
b) Unemployed
c) Salaried/Signed employment
d) Retired/ Prensioner/ Sickness benefit
e) Self-employed
f) Not informed

CC BY

Household income
a) Up to 1 minimum wage
b) 1 to 2 minimum wages
c) 3 to 4 minimum wages
d) Greater than 4 minimum wages
e) No income
f) Not informed

Personal income
a) Up to 1 minimum wage
b) 1 to 2 minimum wages
c) 3 to 4 minimum wages
d) Greater than 4 minimum wages
e) No income
f) Not informed

Level of education
a) Illiterate
b) Elementary education complete/incomplete
c) High school education complete/incomplete
d) Higher education complete/incomplete
e) Not informed

Have you ever received or taken blood (blood transfusion)?
a) Yes
b) No
c) Unable to inform

Anyone in the Family with HTLV?
a) Yes
b) No
c) Unable to inform

If so, who:
a) Father
b) Mother
c) Partner
d) Children
e) Siblings

Do you have children?
a) Yes
b) No

Number of children:

Did you breastfeed your children ?
a) No
b) Yes
c) No, it's a man
d) Not informed

City where you live (residence)
a) Vitória da Conquista
b) Other
c) Not informed

If (b): Which city:

Housing zone
a) Urban area
b) Rural area

Neighborhood:

Housing situation
a) Lives alone
b) Lives with Family
c) Lives with others (friends, etc)
d) Not informed

How many people live in the same house?
a) Uo to 3 people
b) 3-5 people
c) More than 5 peolple
d) Not informed

Do you have running water?
a) Yes
b) No
c) Not informed

Do you have garbage collection?
a) Yes
b) No
c) Not informed

Do you have sewage system?
a) Yes
b) No
c) Not informed

Power grid?
a) Yes
b) No
c) Not informed

Physical activity
a) Yes
b) No
c) Not informed

If so, how many times a week?

Use of illicit substances
a) Yes
b) No
c) Not informed

If so, which ones?
a) Marijuana
b) Cocaine
c) Crack
d) ECSTASY/LSD
e) Other

Number of sexual partners in your entire life
a) <5
b) 5-20
c) >20
d) Not informed

Use of condoms
a) Consistent usage (always)
b) Incosistent usage
c) Not reported
